# Inosine-Containing RNA Is a Novel Innate Immune Recognition Element and Reduces RSV Infection

**DOI:** 10.1371/journal.pone.0026463

**Published:** 2011-10-18

**Authors:** Jie-ying Liao, Sheetal A. Thakur, Zachary B. Zalinger, Kevin E. Gerrish, Farhad Imani

**Affiliations:** 1 Laboratory of Respiratory Biology, National Institute of Environmental Health Sciences, National Institutes of Health, Durham, North Carolina, United States of America; 2 Gene Array Core Facility, National Institute of Environmental Health Sciences, National Institutes of Health, Durham, North Carolina, United States of America; 3 ViraSource Laboratories, Durham, North Carolina, United States of America; McMaster University, Canada

## Abstract

During viral infections, single- and double-stranded RNA (ssRNA and dsRNA) are recognized by the host and induce innate immune responses. The cellular enzyme ADAR-1 (adenosine deaminase acting on RNA-1) activation in virally infected cells leads to presence of inosine-containing RNA (Ino-RNA). Here we report that ss-Ino-RNA is a novel viral recognition element. We synthesized unmodified ssRNA and ssRNA that had 6% to16% inosine residues. The results showed that in primary human cells, or in mice, 10% ss-Ino-RNA rapidly and potently induced a significant increase in inflammatory cytokines, such as interferon (IFN)-β (35 fold), tumor necrosis factor (TNF)-α (9.7 fold), and interleukin (IL)-6 (11.3 fold) (p<0.01). Flow cytometry data revealed a corresponding 4-fold increase in influx of neutrophils into the lungs by ss-Ino-RNA treatment. In our *in vitro* experiments, treatment of epithelial cells with ss-Ino-RNA reduced replication of respiratory syncytial virus (RSV). Interestingly, RNA structural analysis showed that ss-Ino-RNA had increased formation of secondary structures. Our data further revealed that extracellular ss-Ino-RNA was taken up by scavenger receptor class-A (SR-A) which activated downstream MAP Kinase pathways through Toll-like receptor 3 (TLR3) and dsRNA-activated protein kinase (PKR). Our data suggests that ss-Ino-RNA is an as yet undescribed virus-associated innate immune stimulus.

## Introduction

Inflammatory responses that are generated during viral infections are critical for antiviral immune responses. The exact viral recognition elements that activate cells to induce pro-inflammatory signals are not completely characterized. Viral recognition elements such as dsRNA and 5′-triphosphate single-stranded RNA are recognized by several cellular pathways including scavenger receptor class-A (SR-A), dsRNA-activated protein kinase (PKR), retinoic acid inducible gene-1 (RIG-I), Toll-like receptor 3 (TLR3), melanoma differentiation-associated antigen 5 (MDA5), NLR family, pyrin domain-containing 3 (NLRP3) and mitochondrial antiviral signaling proteins (MAVS) [1]–[7]. The intracellular or extracellular interaction of cells with viral recognition elements results in activation of innate immune responses as indicated by expression of inflammatory cytokines and chemokines [1]–[4], [7]–[11].

In addition to the innate immune inflammation, viral recognition elements trigger establishment of an antiviral state, under which each cell resists viral infection. The resistance to viral infection is in part through inhibition of viral replication by perturbation of RNA and protein synthesis. Determining the exact mechanisms by which immune and antiviral responses are activated is essential for understanding viral pathogenesis.

Based on previous data published by our laboratory and others, we hypothesized that any ssRNA, of cellular or viral origin, modified by the presence of inosines (Ino-RNA) was an innate recognition element. RNA with high inosine content is not commonly found in normally growing eukaryotic cells but it is present during infections with DNA and RNA viruses such as polyomavirus, Rous-associated virus, vesicular stomatitis virus, measles virus, and respiratory syncytial virus [12]–[19]. During virus infections, the occurrence of inosines in RNA is by activation of the enzyme ADAR-1 (adenosine deaminase that acts on RNA-1). Once activated, ADAR-1 deaminates adenines into inosines, also termed A-I editing [20]–[25], which occurs in both viral and cellular RNAs. Although the percentage on A-I editing during infection varies, there is evidence for high inosine content within the infected cells [14], [20], [22], [25]–[28]. In fact, based on percentage of adenosine conversion, there are two types of A-I editing. Highly selective deamination shows less than 10% adenosine deamination, While less selective deamination, also called hypermutation, produce A-I editing with more than 10% and often as high as 50% of all adenines can be converted to inosines, which leads to approximately 12.5% inosine content in the RNA [14], [23], [29].

Extracellular Ino-RNA is generated during viral infections. Cell lysis occurs frequently during viral infections, which results in the release of cell content, including intracellular generated Ino-RNA, into the extracellular space. Extracellular dsRNA has been shown to be able to stimulate antiviral responses in neighboring, uninfected cells [30]. However, the role of extracellular Ino-RNA is currently not clear.

Here, using RSV infection as a model, we report that the presence of inosines in ssRNA is a potent inducer of inflammatory cytokines and the antiviral state during virus infection. Our studies showed that in PHBE cells, primary human alveolar macrophages, or C57BL/6 mice, treatment with Ino-RNA significantly induced interferon (IFN-β), tumor necrosis factor (TNF-α), and interleukin (IL-6). Furthermore, treatment of epithelial cells with Ino-RNA led to a reduction in RSV replication. Structural analysis revealed that the presence of inosines increased formation of secondary structures. Extracellular Ino-RNA was internalized by SR-A-mediated endocytosis, which activated downstream MAP kinase pathways through the well-characterized dsRNA specific proteins, TLR3 and PKR. Based on our studies, we suggest that Ino-RNA, of viral or cellular origin, in the surrounding tissue after release from infected cells is a signal for the presence of virus infections.

## Materials and Methods

### Cell culture and virus infection

Primary human bronchial epithelial cell (PHBE) were grown in bronchial epithelial basal medium (BEBM) supplemented with growth factors (Lonza Walkersville, Walkersville, MD, USA) at 37°C in a 5% CO_2_ humidified chamber. BEAS-2B bronchial epithelial cell line was maintained in Dulbecco's Modification of Eagle's Medium/Ham's F-12 with 5% fetal calf serum. Cells, at 90% confluence, were treated with different RNA preparations and total cellular RNA or proteins were then extracted at indicated times.

Isolation of primary human alveolar macrophages was approved by a written consent by the University of North Carolina School of Medicine Committee on the protection of the rights of human subjects. Healthy, non-smoking male volunteers, 18 to 40 year of age, underwent fiberoptic bronchoscopy with lavage to procure human alveolar macrophages. Samples were put on ice immediately after aspiration and centrifuged at 300 x g for 10 min at 4°C. Cells were washed twice with RPMI-1640, and re-suspended in RPMI-1640 at 1×10^6^ cells/ml.

For virus infection, BEAS-2B or PHBE cells were seeded and allowed to reach 70% confluency. Cells were then treated for 10% Ino-RNA, N-RNA, or IFN-α. After 24 hr, cells were infected with respiratory syncytial virus (RSV-A2) at multiplicity of infection (MOI) of 0.1 plaque forming unit (PFU)/cell. Following infection for 24 hr, cells were harvested for total RNA extraction.

Viral titers were measured by standard plaque assay using HEp-2 cells. Plaques were visualized by using goat anti-RSV antibody (Fitzgerald Industries International, Acton, MA, USA) and HRP-conjugated anti-goat secondary antibody (Sigma Chemicals, St. Louis, MO, USA).

### *In vitro* RNA synthesis

pGEM-Luc (p-Luc) vector (Promega, Madison, WI, USA), was linearized with HindIII and purified by Wizard DNA Cleanup kit (Promega, Madison, WI, USA). Run-off transcription of normal p-Luc RNA (transcript a) was performed using the Megascript T7 kit (Ambion, Austin, TX, USA) according to the manufacturer's protocol with nucleotide triphosphates at 7.5 mM each. p-Luc RNA containing 6% Inosine (transcript b) was transcribed by ATP, GTP, CTP and UTP concentrations to 5.6 mM each, and including 9.4 mM ITP in the reaction. RNA containing 10–11% Inosine (transcript c) was transcribed by adjusting ATP, GTP, CTP and UTP concentrations to 2.8 mM each, and including 9.4 mM ITP in the reaction. RNA containing 16% Inosine (transcript d) was transcribed by further reducing GTP concentration to 1.0 mM, where ATP, UTP, and CTP were kept at 2.8 mM each, ITP concentration was kept at 9.4 mM. All transcription reactions were performed at 37°C for 2 hr, followed by digestion of the template DNA and purification using Megascript purification kit according to manufacturer's protocol (Ambion, Austin, TX, USA). The transcripts were then quantitated by UV absorbance, and were analyzed by 1% agarose gel electrophoresis.

### Inosine content analysis

Transcripts were characterized for inosine content by digestion of 3.5 mg of transcript with 2 units each of snake venom phosphodiesterase and calf Intestinal alkaline phosphatase in buffer containing 10 mM Tris pH 8.8, and 2 mM MgCl_2_ (40 ml total reaction volume) for 2 hr at 37°C. Digested products were analyzed by high performance liquid chromotography (HPLC) using C-18 reverse phase column. Relative nucleoside composition was determined by peak integration using Breeze software (Waters Corporation, Milford, MA, USA), and is represented as a ratio of area under each nucleoside peak.

### RNA extraction and RT-PCR

TRIzol total RNA isolation reagent (Invitrogen, Carlsbad, CA, USA) was used for RNA extraction. First strand cDNA was synthesized using superscript reverse transcriptase (Invitrogen, Carlsbad, CA, USA). cDNA synthesis was performed using 0.5 µg total RNA in reverse transcription reaction. Following reverse transcription, 2 µl of cDNA was amplified by RT-PCR. Each experiment was performed in duplicate in 96 well plates using 1 X Sybr Green master mix (Bio-Rad, Hercules, CA, USA) in a final volume of 25 µl. The statistical analysis was performed using parametric two-tailed unpaired t-test with Welch's correction. The sequences of primers were as follows:

IFN-β forward, CAGCAGTTCCAGAAGGAGGA

IFN-β reverse, AGCCAGTGCTCGATGAATCT

TNF-α forward, GGAGAAGGGTGACCGACTCA

TNF-α reverse, TGCCCAGACTCGGCAAAG

IL-6 forward, ATT CTG CGC ACG TTT AAG GA

IL-6 reverse, ATC TGA GGT GCC CAT GCT AC

IL-8 forward, tctggcaaccctagtctgct

IL-8 reverse, gcttccacatgtcctcacaa

RANTES forward, TACCATGAAGGTCTCCGC

RANTES reverse, GACAAAGACGACTGCTGG

RSV-NS1 forward, CACAAACACAATGCCATTCA

RSV-NS1 reverse, AGAGATGGGCAGCAATTCAT

GAPDH forward, GGACCTGACCTGCCGTCTAG

GAPDH reverse, TAGCCCAGGATGCCCTTGAG

### Cell extraction, protein kinase assay, protein purification and western blot analysis

For total cellular protein isolation, PHBE cells were washed 2X in phosphate-buffered saline (PBS) and equal numbers of cells were lysed using 1X SDS-sample buffer with 2.5% β-mercaptoethanol. Prior to electrophoresis, proteins were denatured by heating the samples at 95°C for 5 min. To shear the chromosomal DNA, samples were passed through a 26G needle several times. The proteins were then resolved on a 10% SDS-PAGE and were electrotransferred onto nitrocellulose membranes. The immunoblotted proteins were visualized using the enhanced chemiluminescence (ECL) western blot detection system (Amersham, Arlington Heights, IL, USA). For *in vitro* kinase assays, cell extracts from BEAS-2B cells were prepared using a buffer containing nonidet-p40 detergent and were used for *in vitro* PKR activation as previously described with minor modification [31]. His-tagged human PKR, vaccinia virus dsRNA-binding protein E3L, reovirus σ3 protein and Galpha-i protein (as a negative control, a generous gift from Dr. Lutz Birnbaumer, NIEHS) were grown in E.Coli and purified by affinity chromatography.

Signaling antibodis were used according to the manufacturer's instructions. Rabbit antibodies were used to detect p38 MAPK, phospho-p38 MAPK, JNK, phospho-JNK, phospho-eIF-2α, MDA5 and RIG-I (Cell Signaling Technology, Beverly, MA, USA), TLR3 (Sigma Chemicals, St. Louis, MO, USA), PKR and phospho-PKR (Epitomics Inc., Burlingame, CA, USA).

### siRNA knockdown

The sequences targeted by chemically synthesized small interfering RNAs (Thermo Scientific, Asheville, NC, USA) in transient knockdown experiments were as follows:

TLR3 gene, GGTATAGCCAGCTAACTAG

PKR gene, GCAGGGAGTAGTACTTAAATA

RIG-I gene, GGAAGAGGTGCAGTATATT

MDA5 gene, GGTGAAGGAGCAGATTCAG

PHBE cells were transfected with TLR3-siRNA, RIG-I-siRNA, MDA5-siRNA, or control-siRNA (30 nM) using DharmaFECT transfection reagent (Thermo Scientific, Asheville, NC, USA) according to the manufacturer's instructions. 72 hr after the transfection, cells were stimulated with N-RNA or 10% Ino-RNA. Then, cytokine production was investigated at mRNA level and the phosphorylation state of MAPKs was determined by western blot analysis after.

### RNA structure analysis

Non-denaturing gel electrophoresis of RNA was performed using 0.6% agarose gel in the presence or absence of 120 mM KCl, 4 mM MgCl_2_. Loading buffer consisted of the running buffer with 40% sucrose. For Denaturing gel electrophoresis, we used 1% agarose gel with 2% formaldehyde and 10 mM MOPS. Prior to loading, samples were heated for 10 min at 65°C. For RNA structural analysis using fluorescence spectrum we used acridine orange RNA-binding dye. RNA at 5 µg/ml was dissolved in buffer containing 12 mM Hepes (PH 7.5), 120 mM KCl, 4 mM MgCl_2_. Spectral analysis was performed on a SpectraMax Gemini XS (Molecular Devices, Sunnyvale, CA, USA) with excitation at 460 nm and emission maximum at 530 nm.

### Animal experiments and flow cytometry

All experiments were performed in accordance with the Animal Welfare Act and the U.S. Public Health Service Policy on Humane Care and Use of Laboratory Animals after review of the protocol (ASP# 05-53, LRB) by the NIEHS animal care and use committee. 6–10 week old male C57BL/6 mice (The Jackson Labs, Bar Harbor, ME, USA) were used in all experiments. Mice were housed and maintained in specific pathogen-free conditions. For treatments, anesthetized mice were made to aspirate intratracheally N-RNA, Ino-RNA at indicated concentration or vehicle control (PBS). After indicated times, bronchoalveolar lavage (BAL) was performed using phosphate buffered saline. ELISA was then used to measure the levels of cytokines in the BAL fluid. For PKR knockout experiments, 8–12 week old male mice genetically-deficient in PKR and wild-type control C57BL/6/SV129 were used. After 4 hr treatments, lung tissues were harvested for RT-PCR.

For flow cytometry, BAL cells were first treated with rat anti-mouse CD16/CD32 (BD Pharmingen, San Jose, CA, USA) and as immunoglobulin receptor blockers (Jackson ImmunoResearch, West Grove, PA, USA). Then, one microgram of each monoclonal antibody specific to CD11c (APC), Gr-1 (PE-Cy7) and major histocompatibility complex Class II (MHC II)- FITC (eBiosciences, San Diego, CA, USA), were added and incubated for 30 min on ice. Cell acquisition and analysis were performed on a BD LSR-II flow cytometer using FACSDiva software (version 4.1.2, Beckton Dickinson).

### Confocal microscopy

10% Ino-RNA was labeled with Cy3 using Mirus RNA labeling kit (Madison, WI, USA). Labeled RNA was then purified with RNAeasy mini kit (Qiagen, Valencia, CA, USA). Cells were grown in glass-bottom microwell dishes (MatTek Corp., Ashland, MA, USA) and were treated with 10 µg/ml of Cy3-labeled 10% Ino-RNA alone or were first treated with 10 µg/ml of dextran sulfate or feutin (Sigma Chemicals, St. Louis, MO, USA). After 5 min, cells were washed 3 X in PBS, and mounted with mounting medium (Vector Lab, Burlingame, CA, USA).

### Gene array analysis

Gene expression analysis was conducted using Agilent Whole human Genome 4x44 multiplex format oligo arrays (Agilent Technologies, Santa Clara, CA, USA) following the Agilent 1-color microarray-based gene expression analysis protocol. Starting with 500 ng of total RNA, Cy3 labeled cRNA was produced according to manufacturer's protocol. For each sample, 1.65 µg of Cy3 labeled cRNA was fragmented and hybridized for 17 hr in a rotating hybridization oven. Slides were washed and then scanned with an Agilent Scanner. Data was obtained using the Agilent Feature Extraction software (v9.5), using the 1-color defaults for all parameters. The Agilent Feature Extraction Software performed error modeling, adjusting for additive and multiplicative noise. The resulting data were processed using the Rosetta Resolver® system (version 7.2) (Rosetta Biosoftware, Kirkland, WA, USA).

## Results

### *In vitro* synthesis of RNA and inosine quantitation

**Figure 1:**
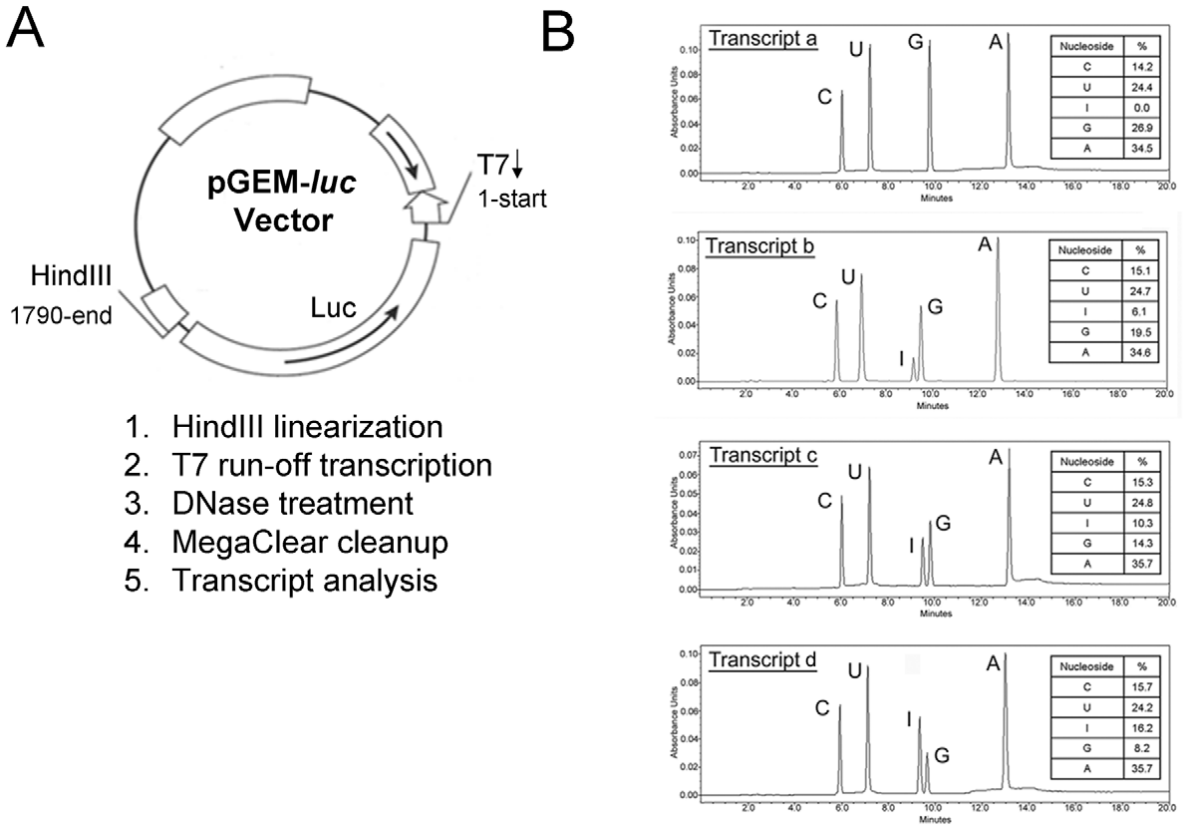
*In vitro* transcription of inosine-containing RNA. RNA was synthesized using Megascript *in vitro* transcription assay (Ambion, Austin, TX, USA) and pGEM-Luc vector (**panel A**) as template (Promega, Madison, WI). For RNA with inosine content, inosine tri-phosphate (Sigma, St. Louis, MO, USA) was added to the reaction mixture. **Panel B**, to quantify incorporation of inosines, RNA was digested by phosphodiesterase and was then separated by HPLC. Relative nucleoside incorporation was determined by calculating the area under each peak. The synthesized RNA is 0% (a), 6% (b), 10% (c) or 16% (d) inosines incorporations.

To examine our hypothesis that the presence of inosines in ssRNA from any source was an innate immune stimulus, we used luciferase gene as an incidental template for *in vitro* synthesis of normal RNA (N-RNA) and inosine-containing RNA (Ino-RNA) (Figure 1). The inosine content of the RNA was then quantified by phosphodiesterase digestion followed by HPLC separation and quantification (Figure 1B). Quantitative analysis showed that we routinely synthesized RNA with 6%, 10% and 16% inosines incorporations, which is similar to A-I editing of RNA during virus infections [14], [23]. Experiments using a second template, Xenopus elongation factor 1α, gave identical results (data not shown).

### Ino-RNA induced inflammatory cytokines and chemokines in human epithelial cells and macrophages

**Figure 2:**
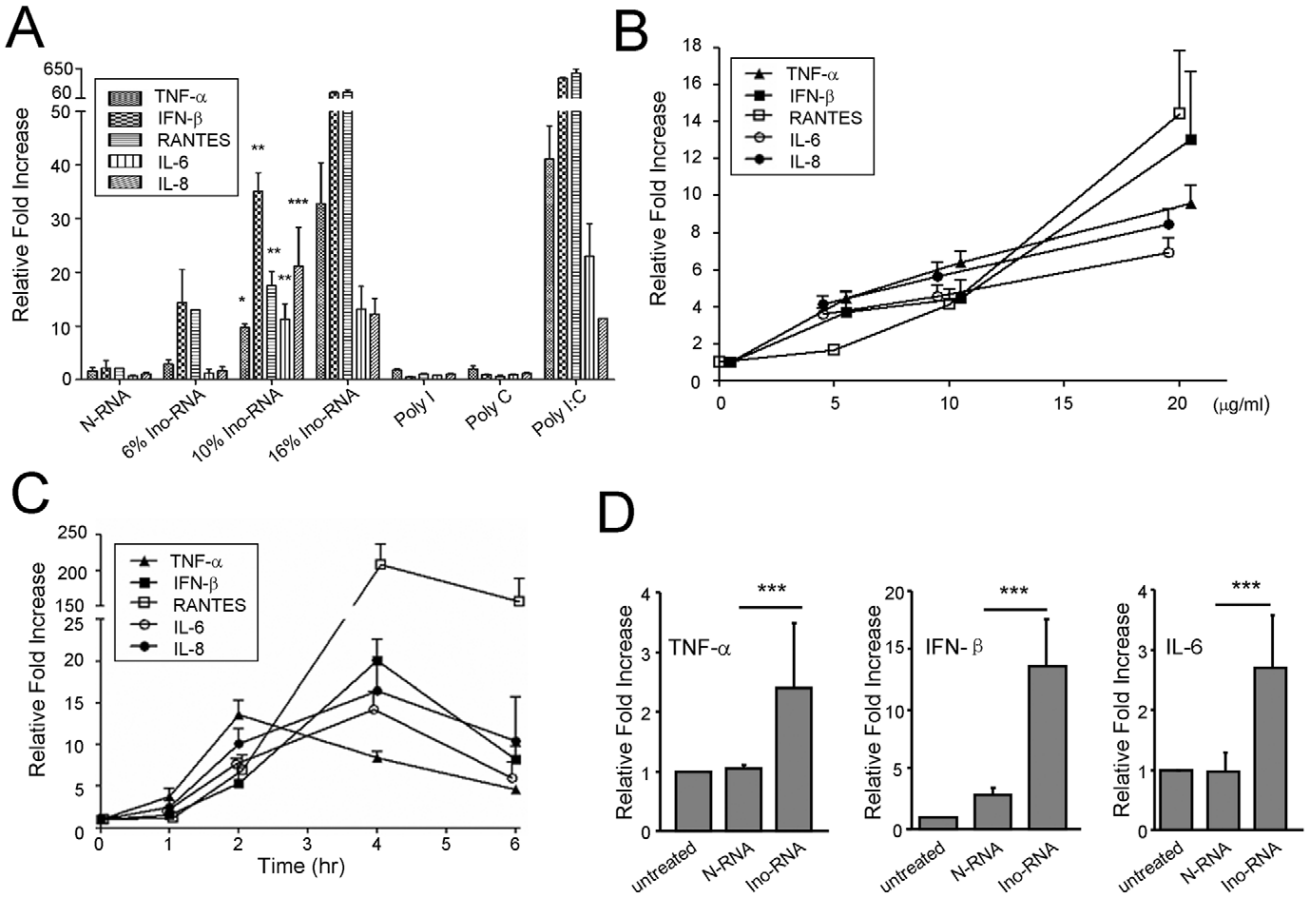
Ino-RNA activates primary human cells. **Panel A**, *In vitro* transcribed N-RNA and Ino-RNA (6%, 10% and 16% inosine content) were added directly to PHBE cells at 10 µg/ml final concentration. After 2 hr, total RNA was extracted and used in real-time RT-PCR (n≥4). We next performed a concentration curve using Ino-RNA with 10% inosine content (2 hr, **panel B,** n = 4) and kinetic studies (**panel C**) analysis of Ino-RNA induction of inflammatory cytokines (n = 4). **Panel D**, treatment of primary human alveolar macrophages with N-RNA and 10% Ino-RNA (n≥4). Error bars indicate standard error of the mean (± SEM, *p<0.05, **p<0.01, ***p<0.001).

To assess the effect of Ino-RNA on innate inflammatory responses, we first treated PHBE cells with N-RNA and Ino-RNA (10 µg/ml) containing 6%, 10% and 16% inosine incorporation (Figure 2A). RT-PCR on total RNA showed that inosine incorporation induced inflammatory cytokines. Treatment of the cells with N-RNA did not show such increase. Interestingly, we also did not observe any significant increase of inflammatory cytokines by the addition of single stranded poly I or poly C. When Poly I∶C (double stranded) was used as a positive control, a significant increase in inflammatory cytokines was observed (Figure 2A). Also, RT-PCR data showed that there was a time- and concentration-dependent induction of inflammatory markers when cells were treated with Ino-RNA (Figure 2B and C). It is interesting to note that the level of TNF-α reached maximum at 2 hr but other cytokines and chemokines continued to increase up to the 4 hr time point. The reason for this difference is not exactly clear, but it may reflect divergent signaling mechanisms between TNF-α and other cytokines and chemokines. Alternatively, there may be an early and specific mechanism to reduce TNF-α expression. Consistent with data from PHBE cells, treatment of primary human alveolar macrophages with 10% Ino-RNA also showed significant induction of TNF-α, IFN-β and IL-6, as compared to treatment with N-RNA (Figure 2D ).

### Ino-RNA treatment reduced RSV replication

Next, we examined whether the induction of inflammatory cytokines resulted in establishment of antiviral state during RSV infection. PHBE and BEAS-2B cells were treated with N-RNA or 10% Ino-RNA (10 µg/ml). After 24 hr, cells were infected with RSV at MOI of 0.1 pfu/cell. Cells were then collected after an additional 24 hr, for total RNA extraction, or 48 hr for plaque assays.

**Figure 3:**
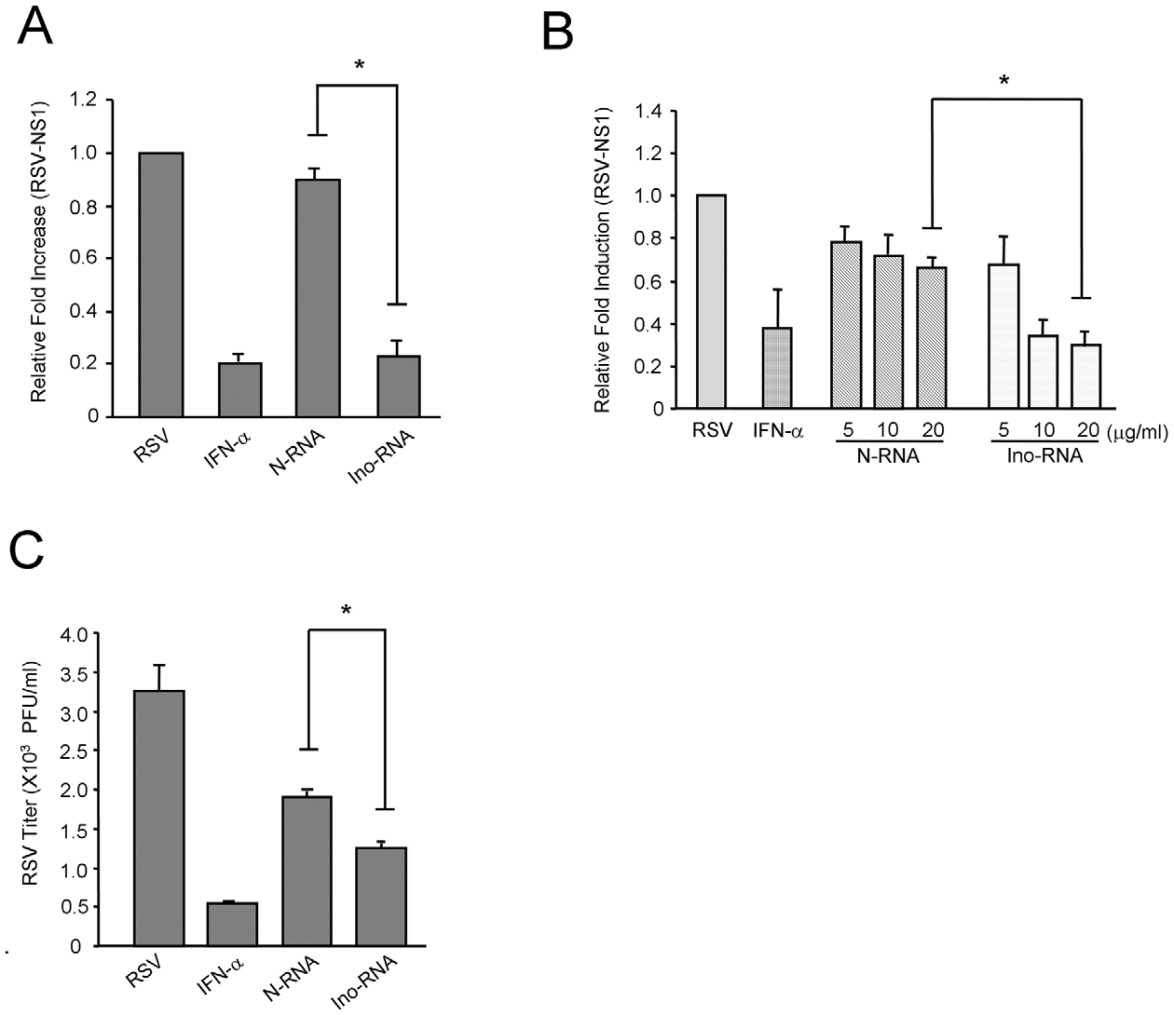
RSV replication is reduced by Ino-RNA treatment. **Panels A**, PHBE cells, were treated with 10% Ino-RNA (20 µg/ml for), N-RNA (20 µg/ml), or IFN-α (1000 Units/ml) for 24 hr prior to RSV infection at 0.1 PFU/cell. Cells were collected after an additional 24 hr, for RNA extraction. Total RNA from infected cells were extracted for RT-PCR quantification of RSV-NS1 transcript (n≥3). **Panel B**, BEAS-2B cells were treated with 10% Ino-RNA (5, 10, 20 µg/ml), N-RNA (5,10, 20 µg/ml) (n≥3). **Panel C**, BEAS-2B cells were treated as above and after 48 hr infection with RSV at MOI of 0.1 PFU/cell, plaque assays were performed (n = 3). Error bars indicate standard error of the mean (± SEM, *p<0.05).

Data in Figure 3A and 3B showed that in both BEAS-2B cell line and in PHBE cells, Ino-RNA treatment significantly reduced viral replication as indicated by reduction in expression of RSV NS1 (non-structural protein-1) gene. The plaque assay, Figure 3C, confirmed the results of RT-PCR assays. These data suggest that Ino-RNA triggers an antiviral state during RSV infection,

### Ino-RNA induces innate immune responses *in vivo*

**Figure 4:**
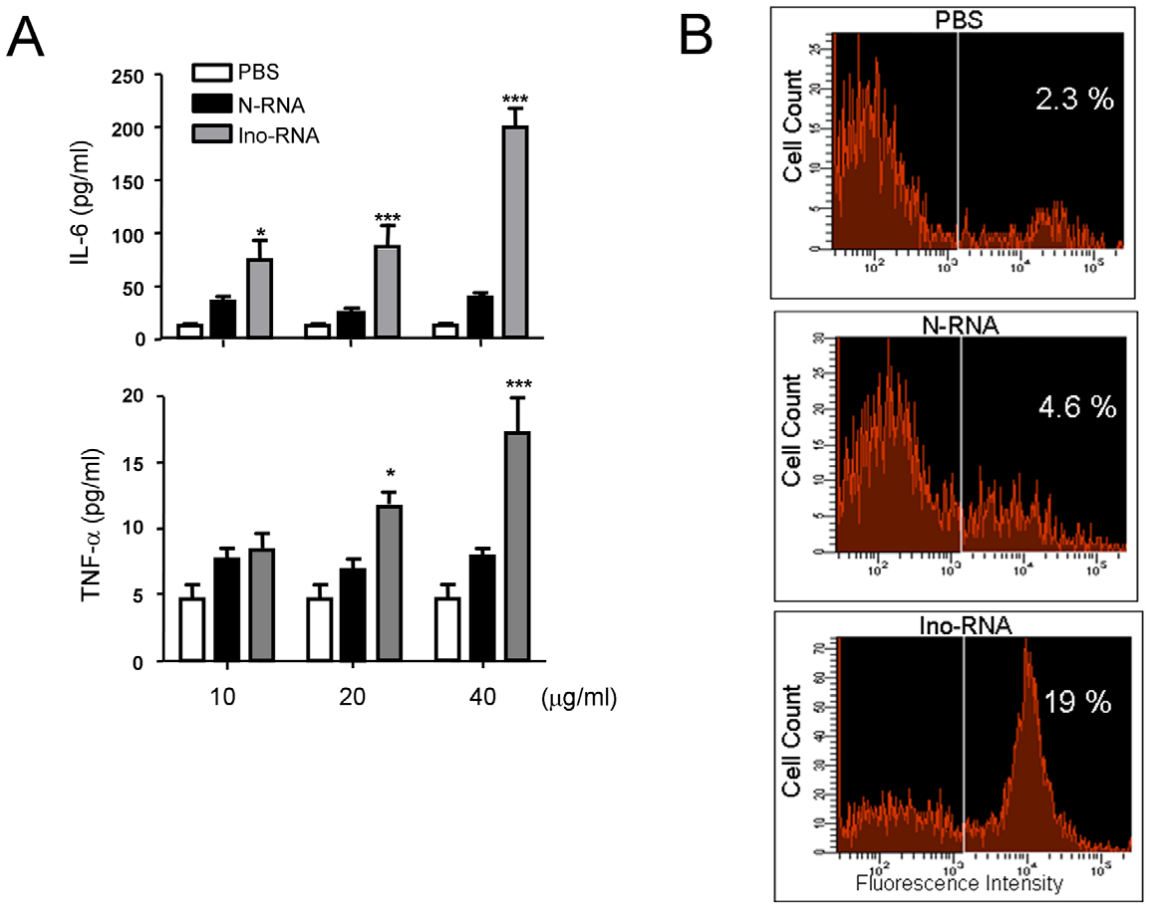
Ino-RNA induces inflammatory responses in C57BL/6 mice. Mice were intratracheally instilled with PBS, N-RNA or 10% Ino-RNA, and BAL fluid was harvested after 24 hr. **Panel A**, cytokine protein levels were determined in BAL fluid by ELISA (n = 3, ± SEM, *p<0.05, *** p<0.001). X-axis presents different RNA concentration. **Panel B**, flow cytometry analysis of neutrophil recruitment in BAL.

To determine the effect of Ino-RNA *in vivo*, we used C57BL/6 mice. Mice were treated with PBS, N-RNA or 10% Ino-RNA. After 24 hr, BAL fluid cells were harvested and used for ELISA and flow cytometry (Figure 4). Results of the ELISA showed that compared to N-RNA, Ino-RNA induced a dose-dependent increase in IL-6 and TNF-α proteins (Figure 4A). We used flow cytometry to test influx of neutrophils into the lungs. As shown in Figure 4B, the increase in N-RNA and Ino-RNA induced influx of neutrophils into the lungs were approximate 2-fold and 8-fold respectively compared to that of PBS treatment. Consistent with our results obtained with human epithelial cells and macrophages, Ino-RNA induced a significant increase in inflammatory cytokines.

### The presence of inosines increases secondary structures of RNA

**Figure 5:**
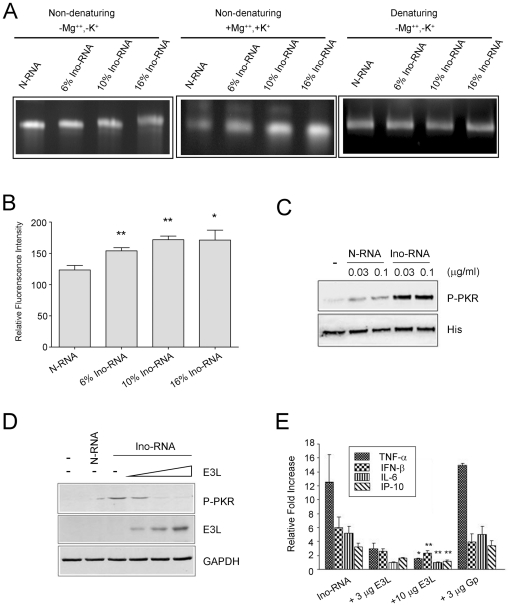
Inosines incorporation increases secondary structures of RNA. **Panel A**, N-RNA, 6%, 10% and 16% Ino-RNA were electrophoresed on non-denaturing or denaturing agarose gels in the absence or presence of MgCl_2_ and KCl. **Panel B**, RNA secondary structures were determined by acridine orange staining in the presence of MgCl_2_ and KCl followed by fluorescence spectral analysis. **Panel C**, Direct interaction of purified recombinant PKR with Ino-RNA was examined by *in vitro* kinase reaction followed by western blot analysis. **Panel D**, BEAS-2B cell extracts were used in *in vitro* kinase reaction in the presence of N-RNA (3 µg/ml), 10% Ino-RNA alone (3 µg/ml) or Ino-RNA incubated with increasing amounts of dsRNA-binding protein E3L at 50, 100 and 200 ng. **Panel E**, PHBE cells were treated with 10% Ino-RNA alone, with Ino-RNA and E3L, Ino-RNA and Galpha-i protein (G_p_) (as a negative control). After 2 hr of incubation, total RNA was collected and RT-PCR reaction was performed (n≥3, ± SEM, *p<0.05, **p<0.01).

We next tested whether inosines incorporation modified the RNA structure. Previous work by others showed that inosines decreased RNA secondary structure [32], [33]. However, since inosine pairing with other nucleotides is promiscuous, we hypothesized that there was a possibility for formation of secondary structures. To test this, we performed RNA structural analysis. First, we used electrophoresis in a non-denaturing agarose gel using Tris-Borate-EDTA (TBE) buffer as the electrolyte. Ino-RNA migrated at a slower rate that N-RNA, suggesting less secondary structure formation (Figure 5A, left panel).

Since a critical factor in RNA structure formation is the presence of cations such as magnesium and potassium, we then excluded ETDA and included MgCl_2_ and KCl in both the electrolyte and the loading buffer at approximate physiologic concentration of 4 mM and 120 mM respectively. The inclusion of cations resulted in a faster migration of Ino-RNA in the non-denaturing agarose gel (Figure 5A, middle panel), despite the fact that inosine has higher molecular weight than the nucleoside it is replacing, namely guanosine. To verify that the faster mobility was due to structural changes, we then used denaturing agarose gel electrophoresis. The data showed that all RNA species ran at similar mobility under denaturing condition (Figure 5A, right panel).

We next used the metachromatic stain acridine orange to study RNA structures. Upon binding to ssRNA acridine orange fluoresces at 640 nm and upon binding to dsRNA the emission is at 530 nm [34], [35]. The fluorescence studies showed that in the presence of magnesium and potassium, Ino-RNA contained more secondary structures (Figure 5B). These results, which are consistent with the results from the non-denaturing agarose gel electrophoresis data, confirm that Ino-RNA contains more secondary structures than N-RNA.

PKR is a dsRNA specific enzyme that is also activated by secondary structures present in ssRNA [31]. To confirm the direct interaction of Ino-RNA with PKR, we performed *in vitro* kinase assays using purified recombinant PKR (Figure 5C) and BEAS-2B cell extract (Figure 5D). Reactions were performed according to materials and methods and autophosphorylation of PKR was detected by western blot analysis. The data in Figure 5C and D showed that the PKR was activated more robustly by Ino-RNA as compared to N-RNA.

To further determine the increased secondary structures of Ino-RNA, we used two different viral proteins that specifically bind dsRNA, namely vaccinia virus E3L and reovirus σ3 proteins [36], [37]. The *in vitro* kinase reactions showed that E3L inhibited Ino-RNA activation of dsRNA binding protein (PKR) (Figure 5D). Similar data was obtained using σ3 protein (data not shown). Furthermore, the inflammatory cytokine induction in PHBE cells by Ino-RNA was significantly inhibited by incubation of Ino-RNA with E3L prior to treatment of the cells (Figure 5E), suggesting that secondary structures in Ino-RNA are required for cell activation.

### Internalization of Ino-RNA is through SR-A-mediated endocytosis

**Figure 6:**
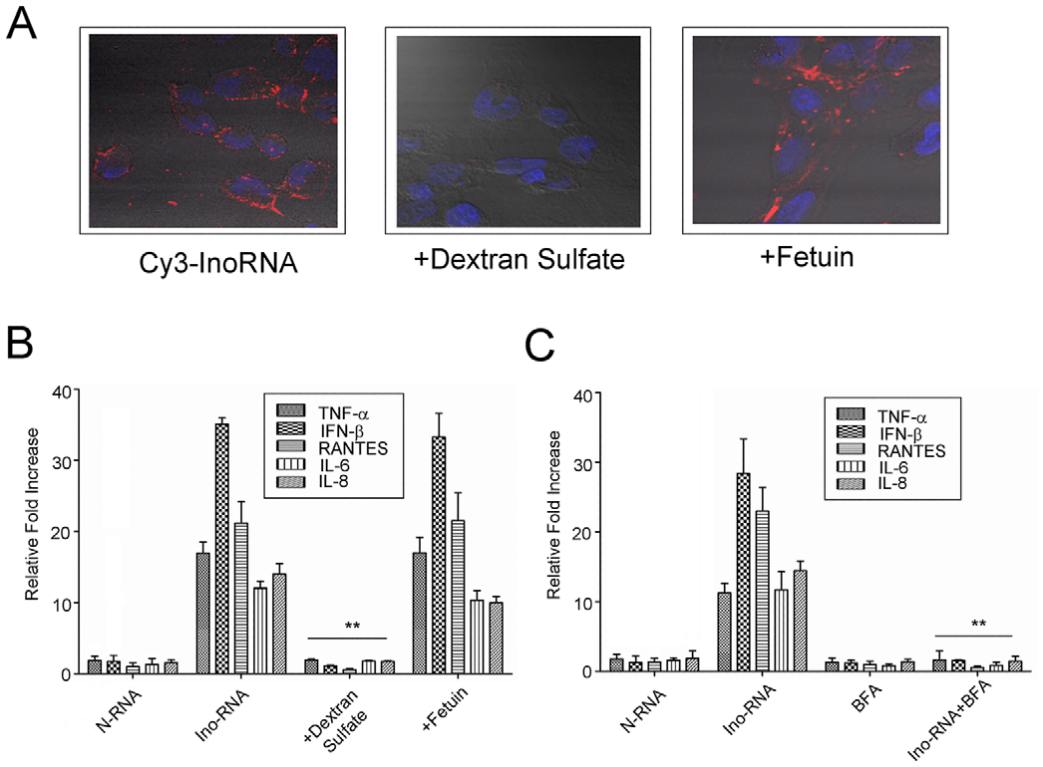
Ino-RNA internalization is through SR-A-mediated endocytosis. **Panel A**, Involvement of scavenger receptors in uptake of 10% Ino-RNA was determined by using dextran sulfate or fetuin. PHBE cells were treated with 10 µg/ml Cy3-labeled Ino-RNA for 5 min, or were treated with 10 µg/ml dextran sulfate or fetuin immediately before addition of Ino-RNA. After 5 min, cells were washed, mounted, and were used in confocal microscopy. **Panel B**, PHBE cells were treated with dextran sulfate or fetuin immediately before treatment with 10% Ino-RNA. After 2 hr total RNA was extracted and used in real-time RT-PCR. **Panel C**, PHBE cells were treated with endosomal acidification inhibitor BFA (100 nM) for 45 min and then 10% Ino-RNA for 2 hr. The total RNA was extracted and used in real-time RT-PCR. Error bars indicate standard error of the mean (n = 3, ± SEM, **p<0.01).

To identify the receptor that is involved in the recognition of Ino-RNA on epithelial cells, we tested SRs as possible candidates. Previous studies from our laboratory showed that SR-A is a cell surface receptor for dsRNA [1]. To visualize the role of SR-A in internalization of Ino-RNA, we used Cy3 fluorescence labeling. After 5 min incubation, cells were washed with PBS and examined by confocal microscopy. In Figure 6A, the data showed that labeled Ino-RNA entry into the cells within 5 min. Furthermore, Ino-RNA was not internalized when cells were treated with competitive antagonists dextran sulfate before the addition of Cy3-labeled Ino-RNA. The data revealed that dextran sulfate, a specific ligand of SR-A, inhibited uptake of Ino-RNA. However, fetuin, a SR-Bspecific ligand, did not decrease Ino-RNA internalization. We further examined the role of scavenger receptors in Ino-RNA induction of inflammatory cytokines. PHBE cells were treated with Ino-RNA in the presence or absence of competitors, and after 2 hr total cellular RNA was harvested and was subjected to real-time RT-PCR (Figure 6B). The data was consistent with that obtained from confocal microscopy. These data suggest that SR-A is involved in the internalization in PHBE cells.

To characterize the downstream pathways, we used the endosomal acidification inhibitor bafilomycine A (BFA), which prevents adequate function of endosomal compartment [38] and recognition by endosomal TLR3 [39]. In Figure 6C, BFA completely blocked cytokine production induced by Ino-RNA. Our data showed that Ino-RNA was recognized by cell surface receptor SR-A and internalized through endocytosis. It was therefore possible that Ino-RNA triggers SR-A-mediated endocytosis and was sensed by TLR3 in endosome.

### Downstream intracellular signaling pathways

**Figure 7:**
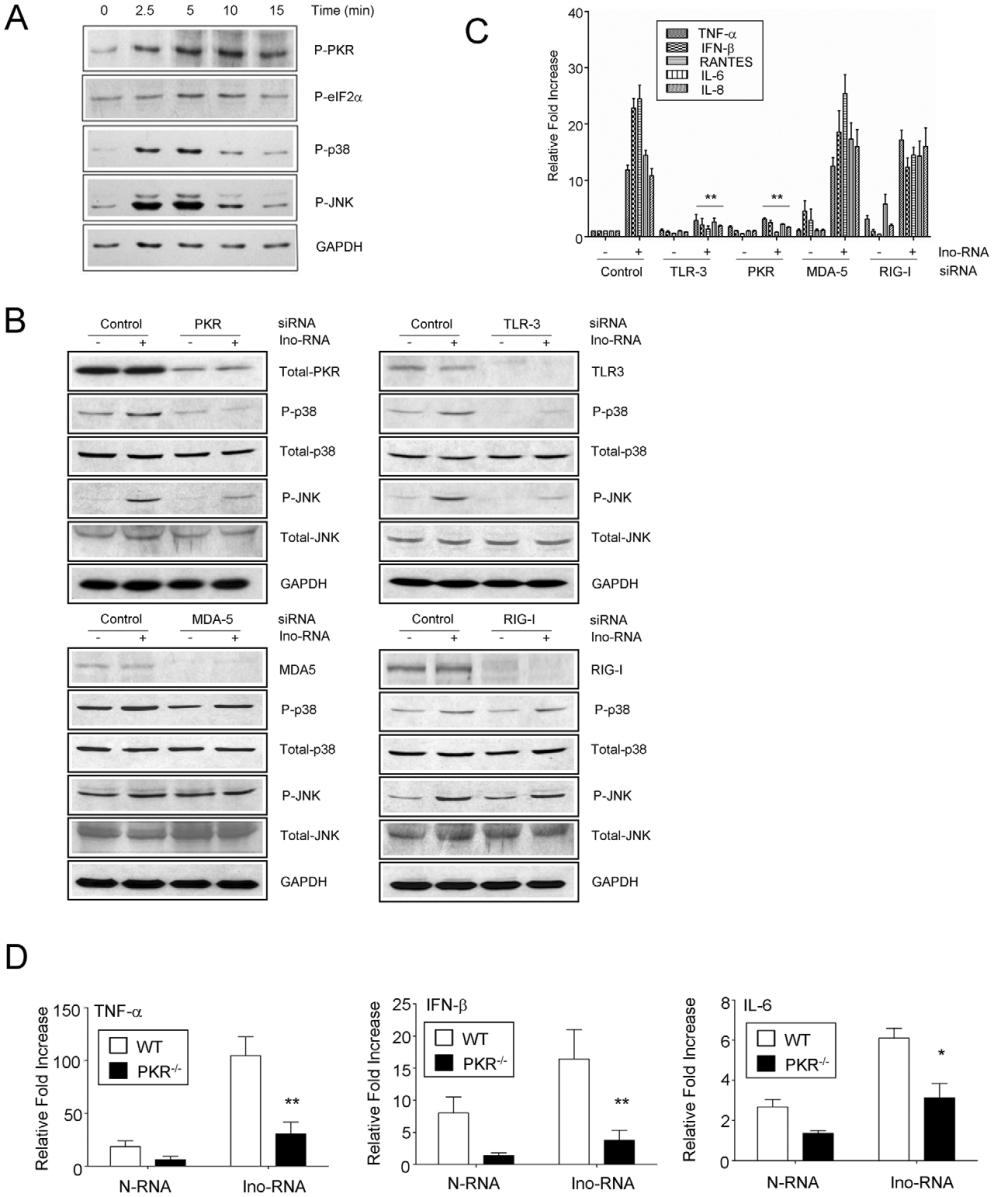
Ino-RNA-induced signaling pathways. **Panel A**, For analysis of innate inflammatory signaling pathways, PHBE cells were treated with Ino-RNA at 10 µg/ml and at indicated time points, total cellular protein extracts were prepared by cell lysis using 1X SDS-PAGE buffer. Phospho-proteins were probed using specific antibodies, and were visualized by the enhanced chemiluminescence detection. **Panel B**, For the activation of MAPKs detection, PKR, TLR3, MDA5 and RIG-I were treated by chemically synthesized siRNAs and then by 10% Ino-RNA. We next performed analysis of Ino-RNA induction of inflammatory cytokines following PKR, TLR3, MDA5 and RIG-I siRNA knockdown (**Panel C**, n = 3). Mock siRNA-treated cells were used as a control. **Panel D**, PKR-deficient mice or wild-type controls (4 in each group, n = 3) were treated intratracheally with 25 µl (1 mg/ml) of Ino-RNA or with 25 µl of PBS (vehicle control). After 4 hr, total lung RNA was isolated for RT-PCR (n = 3, ±SEM, *p<0.05, ** p<0.01).

Since our data showed that Ino-RNA contained more secondary structures, we next determined whether dsRNA-specific signaling pathways could be activated by Ino-RNA. PHBE cells were treated with Ino-RNA at 10 µg/ml and at various times, cell extracts were prepared for western blot analysis. The data showed that there was a rapid activation of dsRNA-specific protein kinase PKR, and the downstream signaling molecules such as eukaryotic initiation factor-2α (eIF-2α), p38 MAPK and JNK (Figure 7A). Treatment of cells with N-RNA did not activate these signaling pathways (data not shown).

Recent reports have shown that TLR3, MDA5 and RIG-I [3]–[5] sense viral RNA to initiate host innate immune responses. To test which of these RNA sensors may be involved in Ino-RNA activated signaling pathway, we transiently knocked down the corresponding proteins using siRNAs. As shown in Figure 7B, the knockdown of TLR3 and PKR, but not that of MDA5 and RIG-I, impaired the activation of p38 and JNK MAPKs in response to Ino-RNA treatment. In identical knockdown experiments, our data showed that TLR3 and PKR but not MDA5 or RIG-I were necessary for inflammatory cytokine induction (Figure 7C).

Our results obtained with the knockdown of PKR provide strong evidence that the PKR protein was an important mediator of MAPK signaling activated by Ino-RNA. To directly evaluate the role of PKR in Ino-RNA induction of immune responses in vivo, PKR^-/-^ and wild type control mice were used. We examined the effect of PKR deficiency on cytokine expression. Total cellular RNA was extracted from lungs and was subjected to RT-PCR. As compared to the wild type, the lungs from PKR^-/-^ mice exhibited significantly diminished expression of TNF-α, IFN-β and IL-6 (Figure 7D).

### Global transcriptomic analysis

**Figure 8:**
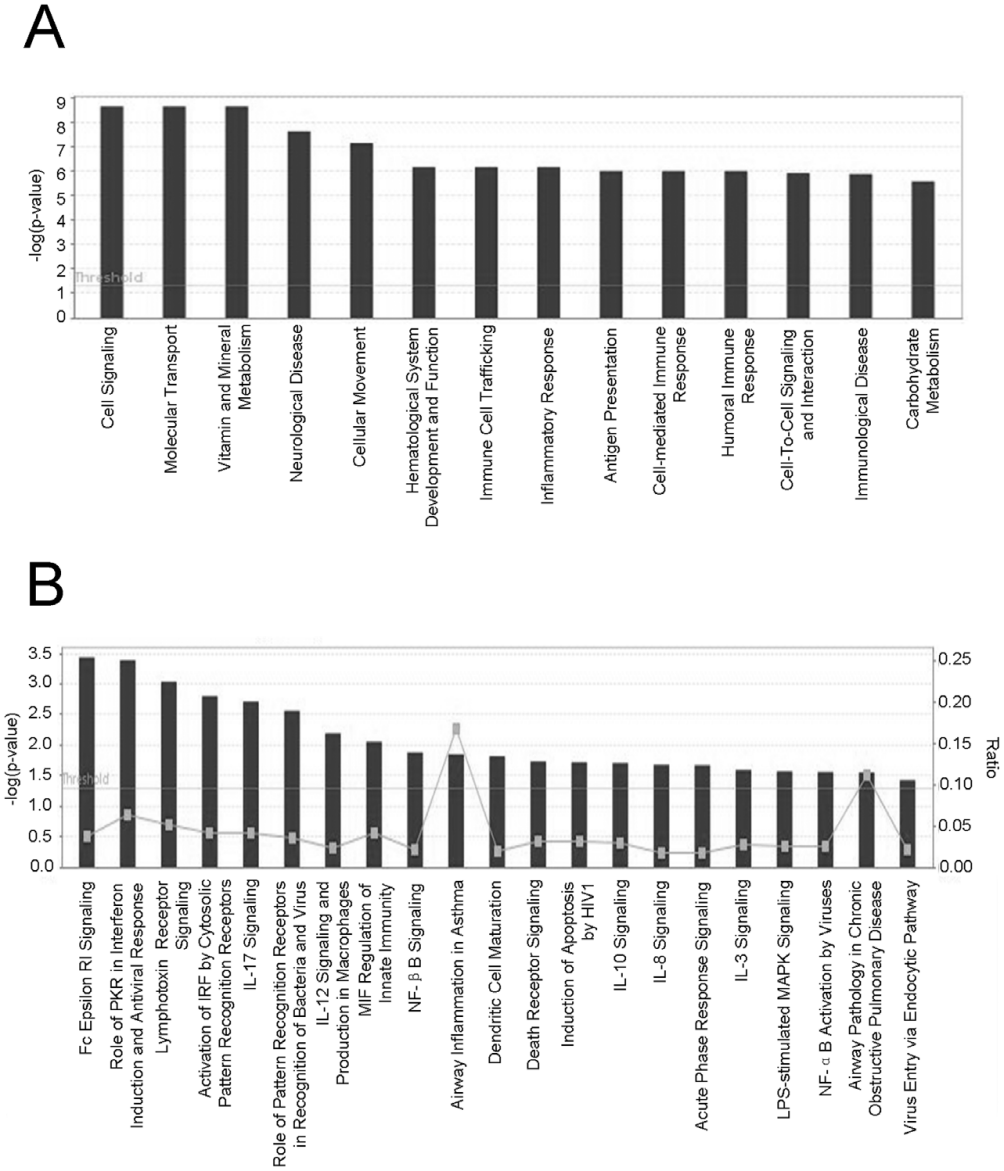
Global transcriptomic analysis. **Panel A**, Ingenuity Bio-Function Analysis of genes upregulated (>2 fold) in 10% Ino-RNA-treated cells but not in normal RNA-treated cells. **Panel B**, Ingenuity Canonical Pathway Analysis of genes upregulated (>2 fold) in Ino-RNA treated cells but not in normal-RNA treated cells.

To identify genes that were induced by Ino-RNA, global gene expression profiling was performed using labeled cRNA derived from total RNA extracted from cells that were treated with N-RNA or with RNA containing 10% inosines. Data were then vigorously filtered to remove biases. The genes that were significantly induced only by 10% Ino-RNA over 2 fold were considered for further analysis. Analysis of our data showed that 10% Ino-RNA treatment induced 132 genes in PHBE cells (Table 1). Genes that are associated with antiviral immune responses are shown in Table 2. Genes that were highly induced by Ino-RNA are associated with antiviral responses, innate and adaptive immunity, and cell signaling (Figure 8). Collectively, these data suggest that Ino-RNA is a potent inducer of innate immune and antiviral genes.

**Table 1:** Microarray analysis of RNA from PHBE cells treated with Ino-RNA.

Treatment	Upregulated genes	Downregulated genes
N-RNA	12	10
Ino-RNA	132	29

Number of genes that were significantly changed over 2 fold in N-RNA-treated cells vs. untreated cells and 10% Ino-RNA-treated cells vs. untreated cells (10 μg/ml) at 2 hr (n = 3).

**Table 2:** List of significant genes associated with antiviral and innate immune responses that are induced by 10% Ino-RNA and not by N-RNA.

Gene	Fold Change	*p*-Value
tumor necrosis factor	15.66287	1.52E-03
superoxide dismutase 2, mitochondrial	7.86062	7.42E-03
interferon-induced protein with tetratricopeptide repeats 2	6.36192	9.00E-05
chemokine (C-X-C motif) ligand 10	5.89583	5.20E-04
lymphotoxin beta (TNF superfamily, member 3)	4.58494	1.30E-04
interferon regulatory factory 1	3.66301	3.63E-03
chemokine (C-X-C motif) ligand 11	3.58219	1.40E-04
complement component 3a receptor 1	3.02745	2.26E-03
Interleukin 7 receptor	2.72687	1.28E-03
nuclear factor of kappa light polypeptide gene enhancer in B-cells inhibitor, alpha	2.71986	5.82E-03
endothelin 1	2.54256	4.70E-04
T-cell leukemia/lymphoma 6	2.50143	5.34E-03
human Alu subfamily J	2.49559	2.18E-03
chemokine (C-X-C motif) ligand 1	2.48883	2.50E-03
protein kinase C, gamma	2.39706	5.42E-03
cathepsin G	2.37645	2.22E-03
serine/arginine repetitive matrix 1	2.33647	3.61E-03
inositol polyphosphate-5-phosphatase	2.28572	9.08E-03
interleukin 8 receptor, alpha	2.08874	8.72E-03
chemokine (C-C motif) ligand 3-like 1	2.06591	1.19E-03
CD5 molecule	2.02828	7.05E-03

## Discussion

In this report we provide evidence to show that extracellular single Ino-RNA is a recognition element for innate immune activation during RSV infections. We demonstrated this by both *in vitro* and *in vivo* experiments using PHBE cells, primary human macrophages and animal experiments (Figure 2 and 4). Previous reports have shown that infected eukaryotic cells recognize viral infections by the presence of different viral RNA structures, such as dsRNA and 5′-triphosphate ssRNA. Recognition of viral RNA structures activates cell signaling through molecules such as SR-A, PKR, TLR3, RIG-I, MDA5, NLRP3 and MAVs [1]–[7]. Our data shows that Ino-RNA has increased secondary structures that activate intracellular signaling pathways and subsequently induced innate inflammatory cytokines. We further report that extracellular Ino-RNA is delivered by SR-A to endosomes and recognized by TLR3 (Figure 6). The downstream MAPK signaling pathway is dependent on TLR3 and PKR but not cytosolic sensors such as MDA5 and RIG-I (Figure 7).

Previous studies by others and our laboratory showed that dsRNA was a critical signal for the presence of viral infections and induction of innate inflammatory responses [10], [11], [40], [41]. However, the amount of dsRNA that is formed during infections has remained controversial [42]. The presence of Ino-RNA during virus infections requires activation of enzyme ADAR-1, a dsRNA-activated adenine deaminase. Therefore, it is possible that small amount of dsRNA present during infections activates this enzyme that subsequently lead to a more abundant Ino-RNA presence. Previous reports have demonstrated activation of this enzyme during infections with several different DNA and RNA viruses, suggesting that dsRNA is present at certain time points during viral life cycles [12]–[19]. Since significant portion of adenosine in cellular and viral RNA can be converted to inosines during viral infections [14], [20], [22], [25]–[28], it is plausible that the presence of abundant intracellular or extracellular Ino-RNA is a potent stimulus for immune activation. Our data obtained from *in vitro* and *in vivo* experiments confirmed this assertion (Figure 2 and 4).

Interestingly, the previous work on activation of cells was nearly entirely done using the synthetic homo-bipolymer poly inosinic∶poly cytidylic acid (poly I∶C). This is likely due to the fact that poly I∶C has been the most potent inducer of type-I interferons, and other ds- or ssRNA polynucleotides have not been as effective [42]. The molecular basis for superior efficiency of poly I∶C over other dsRNA polymers is not yet known. It is tempting to speculate that inosines may be a requirement for efficient activation of innate immunity during virus infection.

Our data, based on four different observations, demonstrated that incorporation of inosine in ssRNA increased secondary structures. First, our data showed that PKR, an intracellular dsRNA-specific kinase was activated by Ino-RNA more than with N-RNA (Figure 5 C and D). We next confirmed the formation of RNA secondary structures using vaccinia virus dsRNA-binding protein E3L. Incubation of Ino-RNA with E3L effectively blocked PKR activation and cytokine expression in PHBE cells (Figure 5E), suggesting that Ino-RNA contained secondary structures. We also reconfirmed this by using the reovirus σ3 dsRNA binding protein (data not shown). Secondly, during agarose gel electrophoresis in the presence of magnesium and potassium Ino-RNA migrated at a faster rate than N-RNA (Figure 5A, middle panel) and the band intensity of Ino-RNA was more than N-RNA. Lastly, the acridine orange spectral analysis showed that incorporation of inosines into RNA increased fluorescence emission (Figure 5B).

A report by Hartner et al. showed that, using ADAR1-deficient mice, ADAR1 was necessary for the maintenance of hematopoietic stem cells and suppression of interferon [43]. They suggested that ADAR-1 has a baseline intrinsic or perhaps secondary function in liver cells. Scadden et al. presented an alternative explanation for the role of ADAR1 in interferon signaling [44]. They proposed that intracellular short IU-pairs in short (20 bp) dsRNA, such as in miRNA suppress interferon induction and apoptosis in cells. In our studies, long (>1 Kb) inosine-containing ssRNA was used which led to an increase in secondary structures and activation of PKR and cytokine expression. These are likely differences due to length of RNA and the possible presence of multiple secondary structures in long inosine-containing RNA. Inosines can pair with any nucleotide and therefore, our data are consistent with the possibility of increased secondary structures after ADAR-1 activation.

Our *in vitro* structural analysis data showing increased secondary structures after inosine incorporation are not consistent with previously published data [32], [33]. This is likely caused by the absence of magnesium and potassium in the other experiments. Cations such as magnesium and potassium are requires for formation and stabilization of RNA secondary structures. In our experiments, data obtained from both electrophoresis and spectral analysis in a buffer lacking magnesium and potassium showed that the Ino-RNA contained less structure than N-RNA (Figure 5A, left panel). However, in the presence of cations, Ino-RNA ran at a faster rate than N-RNA (Figure 5A, middle panel), showing that inosine incorporation increased secondary structure (Figure 5B). Gamper et al. also reported that inosines incorporation into RNA reduced secondary structure in buffer with cations [33]. In their study, transcripts were less than 60 bp long, and inosine content in the RNA was 25%. It is possible that with short transcripts and high inosine content, the secondary structures, are less stable. In our experiments, 1.8 kb transcript with 10% inosine content was used, which is close in size to viral and cellular transcripts. It is known that inosines are promiscuous in their base pairing with other nucleotides, therefore, increased secondary structures in the presence of appropriate cations at physiological concentrations is a very likely event.

It's known that, during virus infection, ADAR activation leads to adenosine to inosine conversion. In our *in vitro* transcription experiment, guanosines triphosphate are replaced by inosine triphosphate (Figure 1), however, we believe that presence of inosines at any sites within RNA will likely increase secondary structures. In addition, we obtained similar results using a different template (Xenopus elongation factor 1 (data not shown).

RNA secondary structures are metastable, nonetheless they can activate innate immune responses. Interestingly, Nallagatla et al. reported that secondary RNA structures capable of activating PKR were also formed in 5′-triphosphate ssRNA [31], and capping of 5′-tri-phosphate RNA abrogated secondary structures and PKR activation. Collectively, the accumulated data demonstrated that formation of secondary RNA structure is a critical factor for viral recognition. Our data in Figure 2 and 3 showed that Ino-RNA induced the expression of TNF-α, IFN-β and the antiviral state. It is known that TNF-α and IFN-β inhibit RSV replication [45]–[48] and our observation that Ino-RNA reduces RSV replication may in part be the result of cytokine expression.

**Figure 9:**
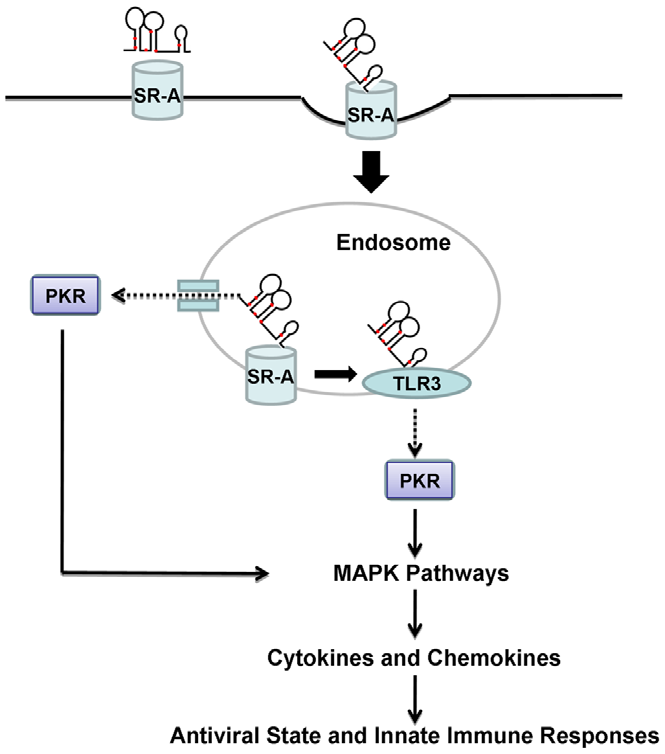
Proposed model of extracellular Ino-RNA-induced antiviral activity.

Previous work showed that SR-A was a surface receptor for extracellular dsRNA [1], [30]. Based on our current data, we propose that SR-A also functions as a surface receptors for extracellular Ino-RNA (Figure 6 A and B). After internalization, Ino-RNA may escape the endosome and interact with PKR directly in the cytoplasm (Figure 5 C and D). It is also possible that Ino-RNA is recognized by TLR3 in the endosomes (Figure 6C) and then activates downstream PKR (Figure 7 B, C and D). Previous study reported that PKR played an important role in TLR3-mediate downstream signaling through interaction with a member of MAP kinase kinase Kinase family [49]. We previously reported that SR-A uptake of dsRNA resulted in downstream PKR activation and MAPK signaling pathways, which induced inflammatory cytokines in epithelial cells [1]. The data in this study further confirm the role of SR-A in Ino-RNA-activated signaling pathways. Knockdown experiments showed that TLR3 and PKR but not MDA5 or RIG-I can significantly reduced p38 and JNK phosphorylation induced by Ino-RNA (Figure 7 B and C). Therefore, we propose that extracellular Ino-RNA-induced antiviral innate immune responses are mediated through PKR and TLR3-dependent pathways (Figure 9). It has been reported that poly I directly added to cells activates B lymphocytes and dendritic cells through a TLR3-dependent and SR-A independent pathway. The differences could be due to different cell [50].

Our global transcriptomic analysis on RNA isolated after 2 hr of treatment showed that 132 genes were preferentially induced by Ino-RNA (Table 1). Several of these genes, such as TNF-α and interferon regulatory factor-1 are relevant to antiviral immune responses (Table 2). It is noteworthy that several cytokines and chemokine receptors were also induced by this treatment, presumably to increase immune cell infiltration into the infected tissue. This is consistent with our *in vivo* data showing an increase in neutrophil infiltration into the lungs of Ino-RNA-treated mice (Figure 4B).

We therefore suggest that fof RNA with secondary structures through an increase in inosines and subsequent activation of intracellular signaling pathway is a critical step in cellular and immune activation.
